# Glycogen phosphorylase inhibitor, 2,3‐bis[(2E)‐3‐(4‐hydroxyphenyl)prop‐2‐enamido] butanedioic acid (BF142), improves baseline insulin secretion of MIN6 insulinoma cells

**DOI:** 10.1371/journal.pone.0236081

**Published:** 2020-09-22

**Authors:** Lilla Nagy, Ferenc Béke, László Juhász, Tünde Kovács, Éva Juhász-Tóth, Tibor Docsa, Attila Tóth, Pál Gergely, László Somsák, Péter Bai

**Affiliations:** 1 Department of Medical Chemistry, Faculty of Medicine, University of Debrecen, Debrecen, Hungary; 2 Department of Organic Chemistry, Faculty of Science and Technology, University of Debrecen, Debrecen, Hungary; 3 Department of Cardiology, Faculty of Medicine, University of Debrecen, Debrecen, Hungary; 4 MTA-DE Lendület Laboratory of Cellular Metabolism, Debrecen, Hungary; 5 Research Center for Molecular Medicine, University of Debrecen, Debrecen, Hungary; Hungarian Academy of Sciences, HUNGARY

## Abstract

Type 2 diabetes mellitus (T2DM), one of the most common metabolic diseases, is characterized by insulin resistance and inadequate insulin secretion of β cells. Glycogen phosphorylase (GP) is the key enzyme in glycogen breakdown, and contributes to hepatic glucose production during fasting or during insulin resistance. Pharmacological GP inhibitors are potential glucose lowering agents, which may be used in T2DM therapy. A natural product isolated from the cultured broth of the fungal strain No. 138354, called 2,3-bis(4-hydroxycinnamoyloxy)glutaric acid (FR258900), was discovered a decade ago. *In vivo* studies showed that FR258900 significantly reduced blood glucose levels in diabetic mice. We previously showed that GP inhibitors can potently enhance the function of β cells. The purpose of this study was to assess whether an analogue of FR258900 can influence β cell function. BF142 (*Meso*-Dimethyl 2,3‐bis[(*E*)‐3‐(4‐acetoxyphenyl)prop‐2‐enamido]butanedioate) treatment activated the glucose-stimulated insulin secretion pathway, as indicated by enhanced glycolysis, increased mitochondrial oxidation, significantly increased ATP production, and elevated calcium influx in MIN6 cells. Furthermore, BF142 induced mTORC1-specific phosphorylation of S6K, increased levels of PDX1 and insulin protein, and increased insulin secretion. Our data suggest that BF142 can influence β cell function and can support the insulin producing ability of β cells.

## Introduction

Diabetes mellitus is the most common endocrine and metabolic disorder. Type 2 diabetes mellitus (T2DM), responsible for 90–95% of diabetes cases, is characterized by hyperglycemia due to a combination of insulin resistance and inadequate compensatory insulin secretion in β cells [1]. Pancreatic β cells are responsible for the synthesis and secretion of insulin, a hormone that maintains normoglycemia by inducing cellular glucose uptake and anabolic processes, such as glycogen synthesis. Consequently, insulin resistance leads to glycogen breakdown and impaired glucose tolerance [2]. Insulin secretion is induced by glucose uptake followed by glucose catabolism via glycolysis and mitochondrial oxidation, which enhances ATP levels. Elevated ATP production leads to depolarization and opening of calcium channels, allowing calcium influx and, subsequently, insulin secretion [3]. Since glucose is the main stimulus for β cell insulin secretion, persistent hyperglycemia combined with peripheral insulin resistance induce β cells to increase their insulin secretion to compensate for high blood glucose levels in T2DM [3–6]. Chronic exposure to high glucose culminates in the exhaustion of β cells, resulting in their depletion and dysfunction [6].

Impaired insulin action leads to high blood glucose levels, mainly from hepatic glucose production (HGP) via glycogen breakdown [1]. Glycogen degradation is catalyzed by the glycogen phosphorylase (GP) enzyme. GP has seven binding sites [7, 8] that are potential targets for allosteric modulation by pharmacological agents [9, 10]. Pharmacological GP inhibitors can be used as glucose lowering agents in the therapy of T2DM [10, 11]. While these studies focused on the hepatic effects of GP inhibition, our previous study provided evidence that GP inhibition can also potently enhance the function of β cells. Structurally different GP inhibitors can induce the synthesis and secretion of insulin, as well as survival signals, in a β cell model [12].

In this study, we investigated BF142, a tartaric acid derived GP inhibitor, which is an analogue of 3-bis(4-hydroxycinnamoyloxy)glutaric acid (FR258900). FR258900 is a natural product isolated from the cultured broth of the fungal strain No. 1383542. *In vivo* studies showed that FR258900 significantly reduced blood glucose levels in db/db mice and STZ-induced diabetic mice [13, 14]. Tartaric acid-derived GP inhibitors were effective at decreasing rabbit muscle GP activity in the low micromolar range, and are thought to bind to the allosteric site of the GP enzyme [15]. We investigated the effects of BF142 in the MIN6 cell line, a well-established model for insulin producing β cells.

## Materials and methods

### Chemicals

**Fig 1:**
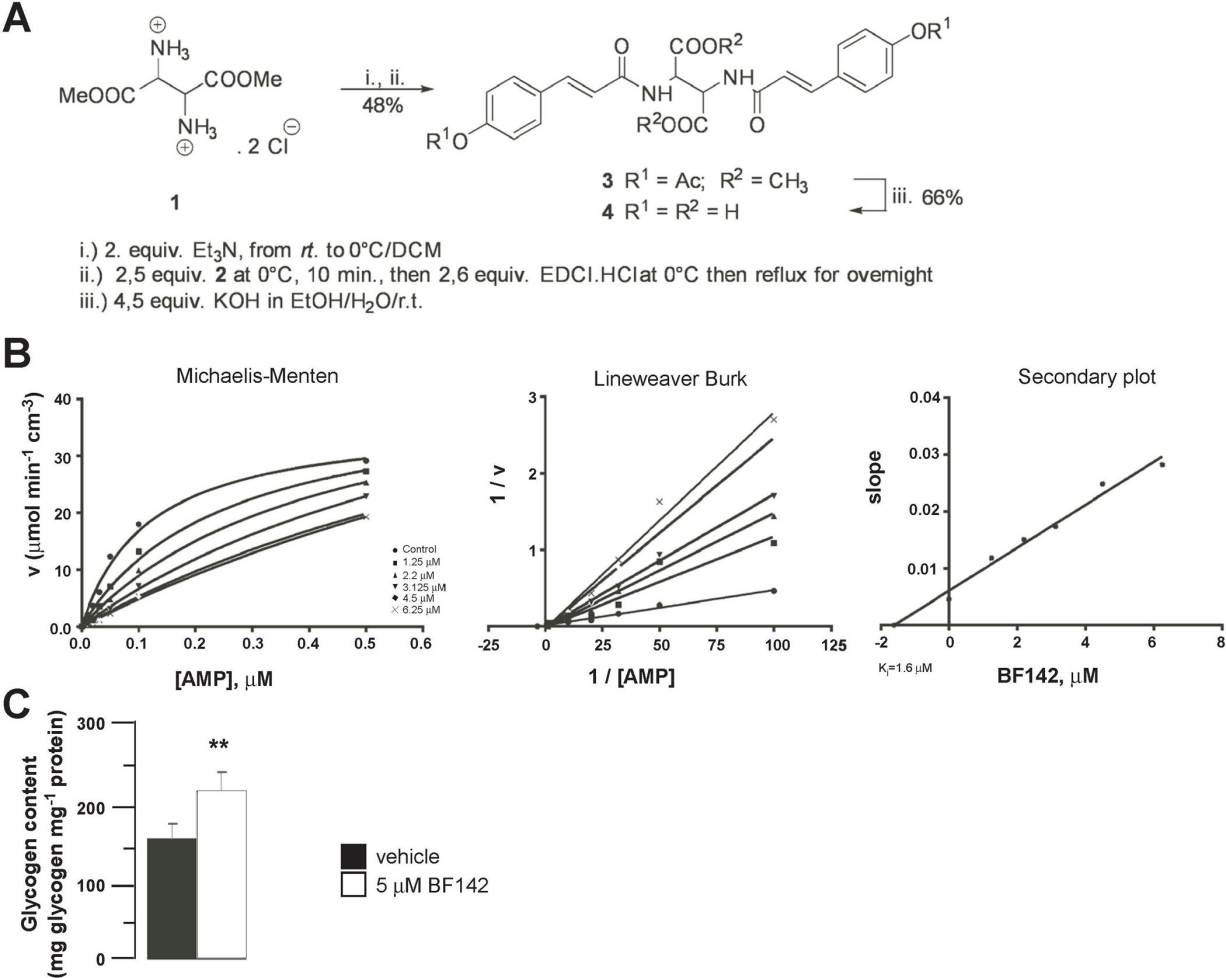
Synthesis of BF142. **(A)** During the synthesis of BF142, the free diamine was liberated from its salt **1** by adding freshly distilled Et_3_N then acylating with *O*-acetylated *p*-coumaric acid [30] **2** using EDCIxHCl as an activating agent [31]. The desired product **3** was isolated in moderate yield (48%). The unprotected carboxylic acid **4** was obtained after hydrolytic deprotection with a good yield (66%). i) 2. equiv. Et_3_N rt. in DCM; ii) 2.5 equiv. **2** at 0°C, 10 min., then 2.6 equiv. EDCIxHCl at 0°C then reflux for overnight; iii) 4.5 equiv. KOH in EtOH/H_2_O/r.t. **(B)** Kinetics of BF142 inhibition of GP were analyzed at a constant concentration of glycogen (1 m/V%), and varying concentrations of AMP (4–40 mM) depicted as the Michaelis-Menten plot. By replotting the slopes of double reciprocal plots against the effective inhibitor concentrations, the secondary plot was generated showing the K_i_ for the inhibitor. **(C)** In BF142-treated MIN6 cells, glycogen content was determined by phenol-sulfuric colorimetric assays (n = 6, in duplicate). ** indicate significance at p<0.01 between vehicle and BF142 groups. Abbreviations in the text.

Unless otherwise stated, all chemicals were purchased from *Sigma-Aldrich* (St. Louis, MO, USA). BF142, the tartaric acid derivative, was synthesized in the Laboratory of László Somsák in the Department of Organic Chemistry, University of Debrecen (**Fig 1A**). BF142 was administered at a concentration (5 μM) close to the K_i_ value (K_i_ = 1.6 μM), to ensure pharmacological specificity.

### Synthesis of BF142

**Fig 2:**
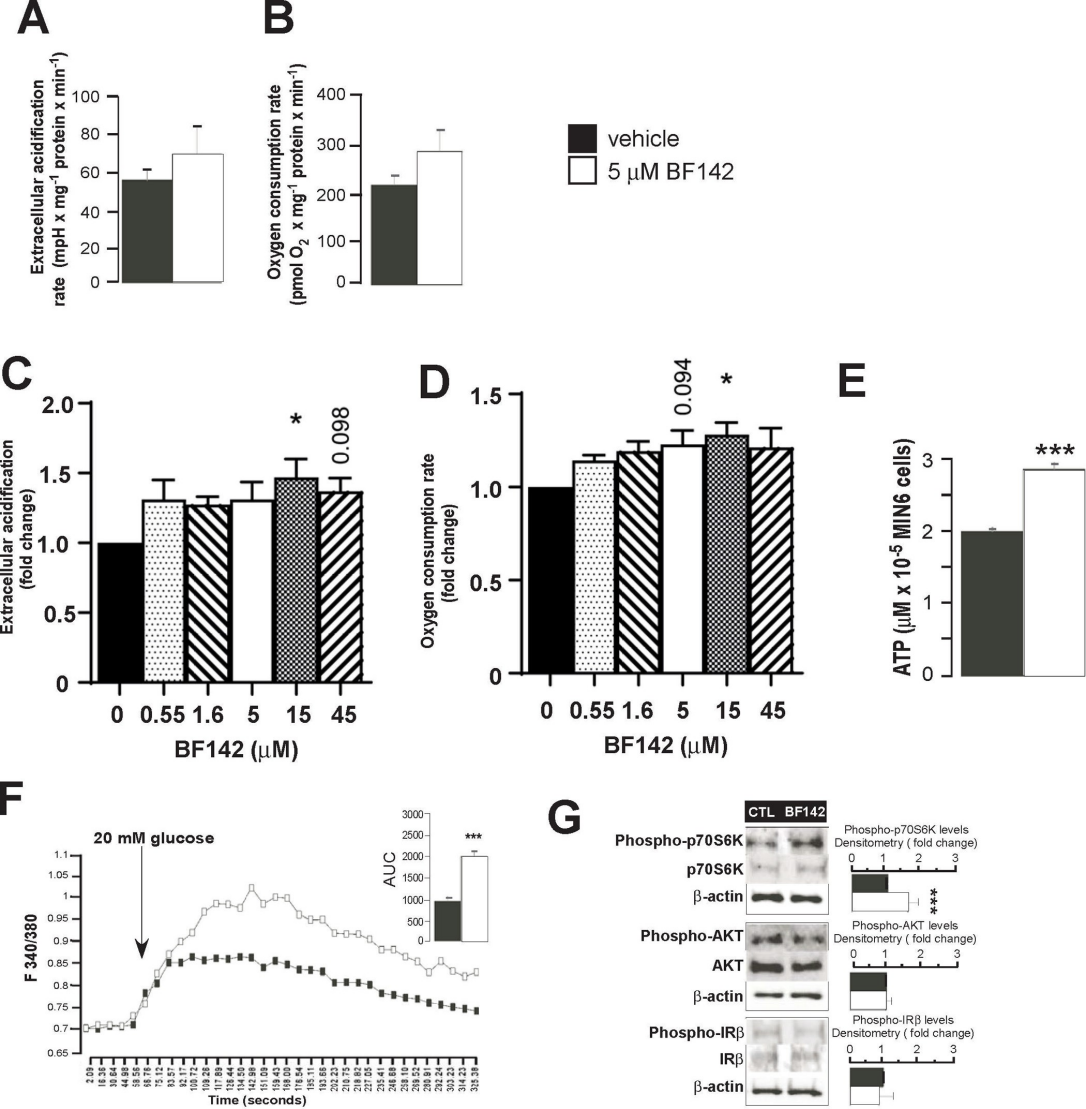
BF142 induces insulin biosynthesis and secretory pathways. MIN6 cells were treated with BF142 for 1 day in the concentrations indicated. In these cells **(A)** extracellular acidification rate (ECAR) (n = 5, in sextuplicate) and **(B)** cellular oxygen consumption rate (OCR) (n = 5, in sextuplicate) were determined using the Seahorse extracellular flux analyzer. The concentration dependency of **(C)** ECAR (n = 3 in octuplicates) and **(D)** OCR (n = 3 in octuplicates) were assessed using a Seahorse flux analyzer. **(E)** ATP concentration was determined in luminometric assays (n = 4, in duplicate). **(F)** Calcium influx was induced by 20 mM glucose and was determined by fura-2AM staining (n = 5, four area of interest/sample; AUC, area under the curve). **(G)** In the same Min6 cells the expression of the indicated proteins were determined by Western blotting. The ratio of the phosphorylated and the total protein were determined by densitometry (n = 5). All abbreviations are in the text. * and *** indicate significance at p<0.05 or p<0.001 between vehicle and BF142 groups, respectively.

**Fig 3:**
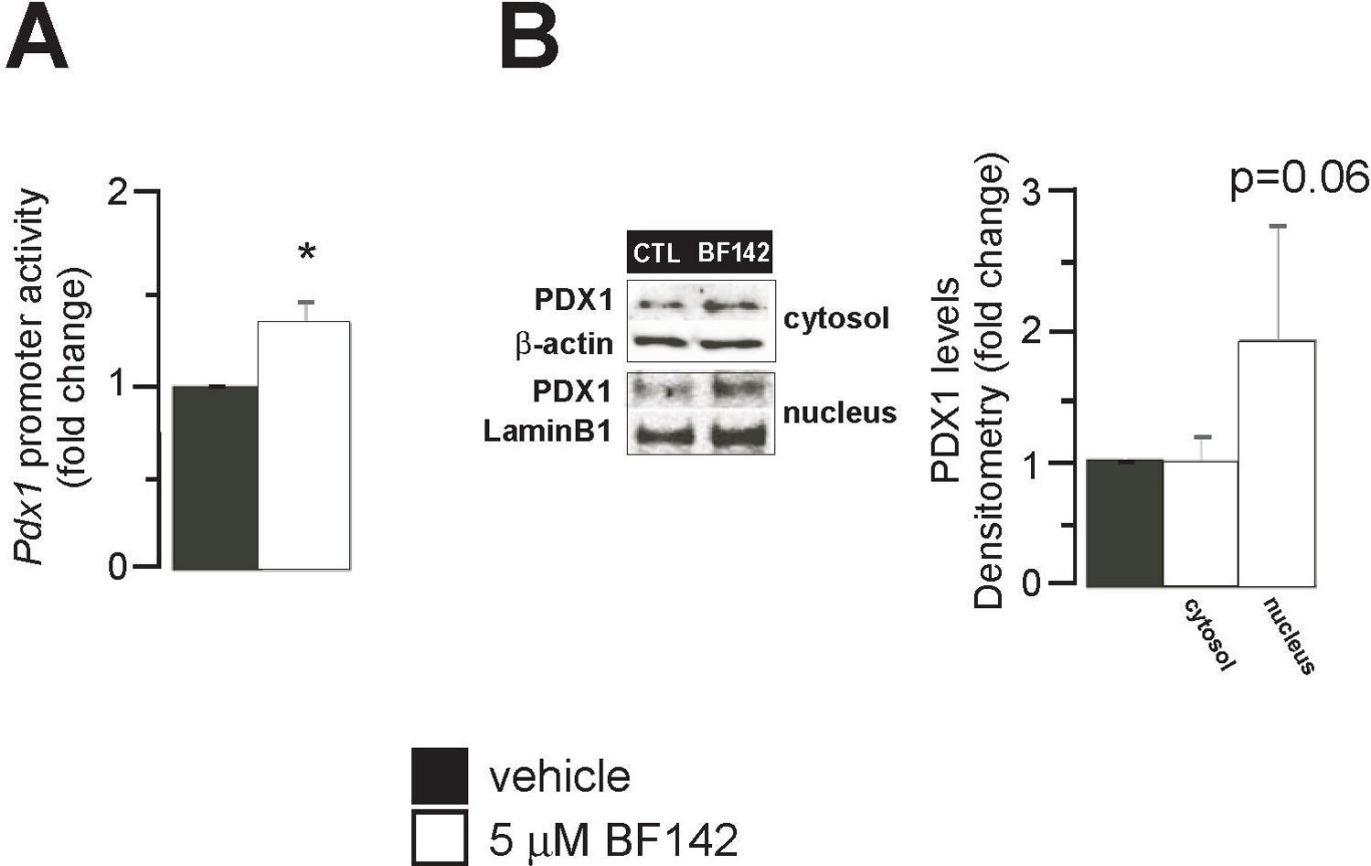
BF142 induces Pdx1 expression. MIN6 cells were treated with BF142 for 1 day. In these cells **(A)** *Pdx1* promoter activity by luciferase assay was determined. **(B)** PDX1 protein levels were determined in the nuclear fractions of MIN6 cell lysates by Western blotting. The number of parallel measurements were 5 in every case (n = 5). All abbreviations are in the text. * indicate significance at p<0.05 between vehicle and BF142 groups.

*Meso*-Dimethyl 2,3‐bis[(*E*)‐3‐(4‐acetoxyphenyl)prop‐2‐enamido]butanedioate (**Figs 1A–3**).

In a flame dried round bottomed flask, *meso-*dimethyl-(2,3-diaminobutanedioatedihydrochloride (500 mg, 2 mmol) was suspended in dry dichloromethane (10 cm^3^; distilled freshly from P_2_O_5_). Subsequently, Et_3_N (0.56 cm^3^, 4 mmol; distilled freshly from KOH) was added to the suspension and the mixture was sonicated. The white suspension was cooled down to 0°C and **2** (1.03 g, 5 mmol) was added. Stirring was continued for 10 min and then EDCI.HCl (1.00 g; 5.2 mmol) was added and the mixture was refluxed overnight. The mixture was then extracted with a saturated aqueous solution of NaHCO_3_ (3 x 10 cm^3^) and water (3 x 10 cm^3^). The organic layer was dried over MgSO_4_. After evaporation of the solvent, the residue was purified by column chromatography (ethyl acetate: hexane = 4:1; R_f_ = 0.25) to give **3** (540 mg, 48%) as a white amorphous solid.

**Fig 4:**
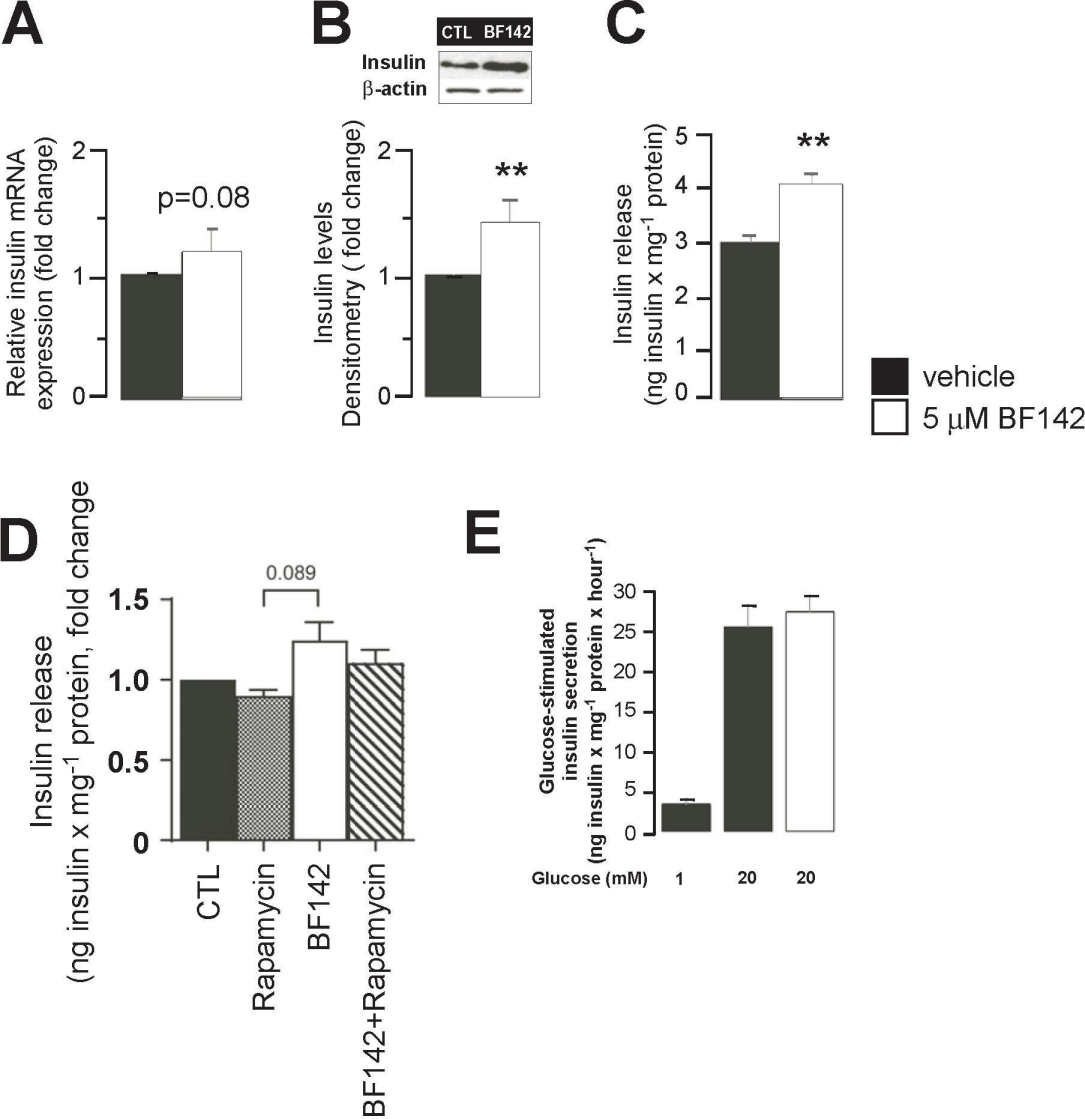
BF142 induces insulin expression and secretion. MIN6 cells were treated with BF142 for 1 day. In control and BF142-treated cells, insulin **(A)** mRNA (n = 5) and **(B)** protein levels (n = 5), as well as, **(C)** spontaneous insulin release were determined (n = 5). **(D)** Min6 cells were treated with 5 μM BF142, 200 nM rapamycin or with the combination of the two drugs for 1 day, then unstimulated insulin secretion was determined (n = 2). **(E)** MIN6 cells were treated with BF142 for 1 day, then glucose-stimulated insulin secretion was determined (n = 5). All abbreviations are in the text. ** indicate significance at p<0.01 between vehicle and BF142 groups.

^1^H NMR (360 MHz, CDCl_3_) δ 7.64 (d, *J* = 15.6 Hz, 2H, H-3’), 7.50 (d, *J* = 8.6 Hz, 4H, H-3”, -5”), 7.42 (d, *J* = 6.7 Hz, 2H, NH), 7.10 (d, *J* = 8.6 Hz, 4H, H-6”, -2”), 6.48 (d, *J* = 15.6 Hz, 2H, H-2’), 5.26 (d, *J* = 6.7 Hz, 2H, H-2,3), 3.79 (s, 6H, OCH_3_), 2.30 (s, 6H, CH_3_). ^13^C NMR (91 MHz, CDCl_3_) δ 169.32 (C = O), 169.23 (ArO**C**OCH_3_), 166.77 (**C**ONH), 151.94 (C-4”), 141.45 (C-3’), 132.24 (C-1”), 129.12 (C-3”, -5”), 122.09 (C-2’), 119.36 (C-3”, -5”), 55.65 (COO**C**H_3_), 53.51 (C-2,3), 21.28 (CH_3_). Anal. calcd for: C_28_H_28_N_2_O_10_ (552.17): C, 60.87; H, 5.11; N, 5.07. Found: C: 60.85; H: 5.10; N: 5.09 2,3‐bis[(2*E*)‐3‐(4‐hydroxyphenyl)prop‐2‐enamido]butanedioic acid (**Figs 1A–4**).

Compound **3** (526 mg, 0.953 mmol) was suspended in ethanol (10 cm^3^), an aqueous solution of KOH was added (1mol/dm^3^; 2,25 cm^3^), and the mixture was stirred at room temperature. The progress of the reaction was monitored by TLC (toluene–acetic acid = 9: 1). When the transformation was complete, the mixture was acidified to pH ~ 5 with glacial acetic acid and the solvent was evaporated. The residue was purified by column chromatography (toluene: acetic acid = 1:2, R_f_ = 0.22) to give **4** as a white amorphous solid (0.279 g, 66%).

^1^H NMR (400 MHz, D_2_O) δ 7.23 (d, *J* = 15.8 Hz, 2H, H-3’), 7.20 (d, *J =* 8.9 Hz, 4H, C-2”, -6”), 6.69 (d, *J* = 8.5 Hz, 4H, H-3”, -5”), 6.30 (d, J = 15.8 Hz, 2H, H-2’), 4.91 (s, 2H, H-2,3). ^13^C NMR (400 MHz, D_2_O) δ 175.61 (**C**OOH), 169.60 (**C**ONH), 158.51 (C-4”), 142.38 (C-3’), 130.90 (C-3”, -5”), 127.66 (C-1”), 118.11 (C-2’), 116.61 (C-2”, 6”), 57.46 (C-2,3). Anal. calcd for: C_22_H_20_N_2_O_8_ (440.12): C, 60.00; H, 4.58; N, 6.36, Found: C: 60.09; H: 4.57; N: 6.39

### Cell culture

MIN6 cells, a generous gift from Dr. J. Miyazaki (Osaka University Medical School, Japan) [16], were cultured in DMEM, 15% fetal calf serum, 1% L-glutamine, 1% penicillin-streptomycin, 50 μM 2-mercaptoethanol, and 25 mM glucose. Treatments were performed in the same media containing 5.5 mM glucose. The measurements took place 1 day after the addition of BF142 (BF142 treatment group marked in white in all figures). The control group, CTL (marked in black in the figures), was treated with 0.01% DMSO in DMEM.

### Determination of inhibitory constant (Ki)

Glycogen breakdown was assayed. Kinetic data were collected using muscle or liver glycogen phosphorylase isoforms in the phosphorylated (activated: GP*a*) and dephosphorylated (GP*b*) isoforms. Kinetic data for the inhibition of phosphorylases were obtained at varying concentrations of D-glucose-1-phosphate and a constant concentration of glycogen. Enzymatic activities are presented in a double-reciprocal plot (Lineweaver-Burk). The plots were analyzed by a non-linear data analysis program. The inhibitor constants (K_i_) were determined by secondary plots, replotting the slopes from the Lineweaver-Burk plot against the inhibitor concentrations. The means of standard errors for all calculated kinetic parameters averaged less than 10% [17, 18].

### Biochemical glycogen determination

The KOH-ethanol glycogen extraction method was used to extract glycogen, and the phenol-sulfuric acid assay was applied to determine glycogen content, as described in [12]. Measurements were normalized to protein content.

### Pdx1 promoter assay

Luciferase reporter assays were performed, as described in [19]. Briefly, 1.5x10^5^ MIN6 cells were seeded in 6-well plates and were treated with BF142 for 1 day. At the same time, cells were transfected with pCMV-βgal plasmid (1 μg) and -6500STF-1luc plasmid [20] (5 μg). Luciferase activity was expressed as luciferase activity/β-galactosidase activity, then the values were transformed to fold increase relative to the control. The -6500STF-1luc plasmid was a generous gift from Dr. M. Montminy (Salk Institute, La Jolla, CA, USA). The promoter insert (^+^68 - ^-^6500 bp) is of rat origin. To assess the promoter insert applicability for murine models, we performed a sequence alignment of the promoter sequences and found 82% identity between the promoter sequence of rat and murine *Pdx1*.

### Protein extraction and Western blotting

Cytoplasmic and nuclear protein extraction were performed as described in [12]. PDX1 was measured in nuclear extracts, and all others proteins were measured in the cytoplasmic fractions. Western blotting was performed as described in [21]. Blots were probed with the antibodies summarized in **Table 1**.

**Table 1:** List of antibodies used in the study.

Primary antibodies:	Supplemental figures			
PDX1 (Cell signaling, 5679)	S1A	rabbit	monoclonal	1:1000
LaminB1 (Cell signaling, 12586S)	S1A	rabbit	monoclonal	1:1000
Insulin (DAKO, Glostrup, Denmark, A0564)	S1B	guinea pig	polyclonal	1:1000
Insulin Receptor β (Cell signaling, 3025)	S2A	rabbit	monoclonal	1:1000
Phospho-Insulin Receptor β (Tyrosine 1345)(Cell signaling, 3026)	S2B	rabbit	monoclonal	1:500
AKT (Sigma Aldrich, 9272)	S2C	rabbit	monoclonal	1:1000
Phospho-AKT (Serine 473) (Cell Signaling, 4060)	S2D	rabbit	monoclonal	1:2000
p70S6K (Cell Signaling, 9205S)	S2E	rabbit	polyclonal	1:1000
Phospho-p70S6K (Threonine 389) (Cell Signaling, 9205S)	S2F	rabbit	polyclonal	1:1000
β-Actin−Peroxidase (Sigma Aldrich, A3854)	S1C	mouse	polyclonal	1:25000
**Secondary antibodies:**				
HRP-linked anti-rabbit IgG (Cell Signaling)		goat	polyclonal	1:2500
Peroxidase conjugated anti-guinea pig IgG (Sigma Aldrich)		goat	polyclonal	1:2000

Signals were developed using enhanced chemiluminescence (ECL) and were captured by Fluorchem FC2 gel documentation system (Alpha Innotech, San Leandro, CA, USA). Densitometry was performed using the Image J software, and the intensity results were calculated as fold change with respect to the control.

### Insulin release in MIN6 cells

MIN6 cells were plated, then pre-incubated in DMEM for 1 day with BF142 or vehicle. The medium was changed in each well to 1 ml of 5.5 mM glucose-content DMEM medium (without adding more glucose) and after 2 minutes 10 μl sample was taken and diluted further in medium for subsequent insulin determination. Spontaneous insulin production of MIN6 cells was measured using a Mouse Insulin ELISA Kit (Mercodia, Winston Salem, N.C., USA) following the manufacturer's instructions. After the assay, 1 M NaOH was added to adherent MIN6 cells in 6-well plates and the total protein content was determined using BCA reagent (Pierce^TM^ BCA Protein Assay Kit, Thermofisher, Walthman, MA, USA). Insulin secretion was normalized to protein content.

### Glucose-stimulated insulin release (GSIS) in MIN6 cells

GSIS from MIN6 cells was determined using a static incubation protocol as in [22] and [12]. MIN6 cells were cultured in 96-well plates until ∼80% confluency, and the medium (with 25 mM glucose) was changed every 48 h. On the day of the experiment, growth medium was removed, and the cells were washed twice with glucose-free HEPES-balanced Krebs-Ringer phosphate buffer (KRBH; 111 mM NaCl, 25 mM NaHCO_3_ (pH 7.4), 4.8 mM KCl, 1.2 mM KH_2_PO_4_, 1.2 mM MgSO_4_, 10 mM HEPES, 2.3 mM CaCl_2_ and 0.1% BSA). Cells were preincubated for 1 h in 5% CO_2_ at 37°C in KRBH supplemented with 1 mM glucose. The pre-incubation medium was removed, and the cells were washed once in glucose-free KRBH. The cells were then incubated for 1 h in 20 mM glucose-containing KRBH in the absence (control, CTL) or presence of BF142. At the same time, a set of control cells were incubated for 1 h in 1 mM glucose-containing KRBH for determination of insulin baseline. For all experiments, incubation medium was collected, spun at 1500 g for 5 min 4°C, and then diluted 20X. Insulin concentration in diluted samples was determined using a Mouse Insulin ELISA Kit (Mercodia, Winston Salem, N.C., USA). After the assay, 1 M NaOH was added to the adherent MIN6 cells and the total protein content was determined using BCA reagent (Pierce^TM^ BCA Protein Assay Kit, Thermofisher, Walthman, MA, USA). Insulin secretion was normalized to protein content.

### Determination of oxygen consumption (OCR), and extracellular acidification rate (ECAR)

OCR and ECAR were determined using the XF96 Flux Analyzer (Agilent Technologies, Santa Clara, CA, USA) with the considerations described in [23, 24]. MIN6 cells were seeded in 96-well assay plates and treated with BF142 for 1 day, followed by oximetry. After recording the baseline, OCR and ECAR were recorded every 3 minutes up to 60 minutes. Antimycin (10 μM) was used for distinguishing the mitochondrial from non-mitochondrial oxygen consumption (Proton leak). The final reading was taken at 1 hour. For protein determination, we used 1 M NaOH, 10 times volume of BCA reagent (Pierce^TM^ BCA Protein Assay Kit, Thermofisher, Walthman, MA, USA), and a plate reader. OCR and ECAR were normalized to protein content and normalized readings were analyzed and plotted. OCR values were expressed as pmol O_2_ x 10^−5^ MIN6 cells x minutes^-1^. ECAR values were expressed as mpH x 10^−5^ MIN6 cells x minutes^-1^.

### Determination of cellular ATP levels

Cellular ATP content was determined using an ATP assay Kit (Sigma), following the manufacturer's instructions. ATP concentration was measured in 96-well assay plates (5 x 10^5^ MIN6 cells / well) by a luminometer (CHAMELEON Multilabel Microplate Reader, Hidex Turbo, Finland).

### Measurement of changes in intracellular Ca^2+^ concentrations

Calcium transients were assessed similar to the protocol described in [25]. MIN6 cells were seeded on glass coverslips (25 mm diameter and 1 mm thickness; Thermo Fisher (Thermo Fisher Scientific Gerhard Menzel B.V. & Co. KG, Braunschweig, Germany)) and treated with BF142 for 1 day. Following BF142 treatment, cells were charged with 5 μM of Fura-2AM fluorescent Ca^2+^ indicator dye (Molecular Probes) for 90–120 minutes. Coverslips were placed into a tissue chamber suitable for fluorescence microscopy. The tissue chamber was filled with DPBS (Invitrogen, containing calcium and magnesium) and placed on the stage of an InCyte IM2 system (Intracellular Imaging Inc., Cincinnati, OH, USA). Cells were illuminated alternatively by 340 and 380 nm light (excitation) and pictures were recorded at a wavelength above 510 nm (emission). Four glucose responsive and clearly distinguishable cells (termed area of interest) for each treatment were selected per region of interest. Changes in intracellular Ca^2+^ concentration were recorded as changes in the ratio of emission/excitation (340/380 nm). After reaching a stable baseline (min. 60 seconds), cells were induced with 20 mM glucose.

### Statistical analysis

All data are represented as average ± SEM, and n denotes the number of experiments. Statistical significance was tested using Student’s t test (unpaired, two-tailed). Statistically significant differences between vehicle and BF142-treated groups at p<0.05, p<0.01, and p<0.001 are indicated by *, **, and ***, respectively.

## Results

### Synthesis and applicability of glycogen phosphorylase inhibitor BF142

BF142 was synthesized as a member of an analogue series [15, 26] of FR258900 isolated by Furukawa and co-workers [13]. The synthesis of **4** (**Fig 1A**) was carried out by acylation of *meso*-2,3-diamino-butandioic acid, which was synthesized from fumaric acid, as published in the literature [27–29]. We determined the K_i_ value of BF142. We used BF142 in a concentration (5 μM) close to the K_i_ (K_i_ = 1.6 μM) (**Fig 1B**). Treatment of MIN6 cells with BF142 increased total glycogen content, suggesting that BF142 is active in this model (**Fig 1C**).

### BF142 induces insulin secretion pathways in Min6 cells

We investigated the effects of BF142 on the pathways that lead to insulin secretion. BF142 treatment tended to increase glycolysis, as indicated by increased ECAR (**Fig 2A**), and mitochondrial oxidation, as indicated by increased OCR (**Fig 2B**), compared to control treatment. The effects of BF142 on OCR and ECAR were dose dependent (**Fig 2C and 2D**). BF142 treatment resulted in significantly increased levels of cellular ATP (**Fig 2E**). In line with these findings, BF142 significantly elevated glucose-induced calcium influx (**Fig 2F**) compared to control treatment. In addition to these, BF142 induced the phosphorylation of S6 kinase on threonine 389 that is prerequisite for insulin synthesis, while did not affect the phosphorylation of Akt on serine 473 or the phosphorylation of the insulin receptor on tyrosine 1345 (**Fig 2G**).

We assessed whether BF142 modulates the expression of pancreatic and duodenal homeobox 1 (Pdx1). BF142 treatment induced the activity of the promoter of Pdx1 (**Fig 3A**) that was converted to increases in the nuclear fraction of Pdx1 protein (**Fig 3B**).

### BF142 induces insulin secretion in MIN6 cells

The above-detailed changes suggested enhanced insulin expression and secretion in Min6 cells upon BF142 treatment. Indeed, the mRNA and protein levels of insulin were boosted by BF142 (**Fig 4A and 4B**). In addition, spontaneous insulin release from MIN6 cells increased after BF142 treatment compared to control treatment (**Fig 4C**). BF142-mediated induction of insulin secretion can be inhibited partially, pointing towards the partial mTORC1-dependincy of the effects of BF142 (**Fig 4D**). Although baseline insulin secretion was increased upon BF142 treatment, glucose-induced insulin release was not affected by BF142 treatment (**Fig 4E**).

## Discussion

Glycogen phosphorylase (GP) has become a validated target to modulate glucose levels in T2DM, and pharmacological GP inhibitors are now considered antidiabetic agents [10, 32]. In this study, we investigated the metabolic effects of a novel GP inhibitor, BF142, on MIN6 cells, a pancreatic β cell model. BF142 is a tartaric acid derivative, an analog of FR258900, which has been tested *in vivo*. FR258900 is a natural product that significantly lowers plasma glucose levels in diabetic mice by inhibiting hepatic glycogen breakdown [13]. These results coincide with our previous *in vivo* studies [33–35] in which we showed that glucose analog GP inhibitors (e.g. KB228 (N-(3,5-dimethyl-benzoyl)-N’-(*β*-D-glucopyranosyl)urea) or TH (glucopyranosylidene-spiro-thiohydantoin)) improved glucose tolerance and increased plasma insulin levels in diabetic rats and mice. Glucose tolerance was likely improved by inducing glucose uptake into the liver and skeletal muscle, and enhancing mitochondrial oxidation. Glucose-based GP inhibitors bind primarily to the catalytic site, inducing dephosphorylation and inhibition of GP; however, these molecules were also reported to bind to the allosteric site of GP [9, 10]. Tartaric acid derived GP inhibitors, such as BF142, presumably bind only to the allosteric site of the GP enzyme [15]. Differences in the mechanism of inhibition may be responsible for differences in the biological effects of the inhibitors.

In our previous study, we identified pancreatic islets of Langerhans as a new target tissue for GP inhibition. In these studies, we used glucose analogue GP inhibitors (KB228 and BEVA335) and a reference inhibitor, indole-2-carboxamide compound (CP-316819). CP-316819, which is in phase II clinical trials, binds to a novel allosteric site on GP [36, 37]. The physiological role of glycogen in pancreatic β cells is emphasized by several studies [38–41]. Glycogen can support insulin release under starvation conditions [42, 43] or contribute to glucotoxicity during chronic exposure of β cells to high glucose concentrations [39, 44]. However, a recent study has questioned the importance of glycogen in β cells [45]. The role of glycogen content in β cells is still not clear, however, we have demonstrated that pharmacological GP inhibition can restore pancreatic β cell function and induce increased β cell number [12].

We showed that GP inhibition, and the consequent increase in cellular glycogen content in MIN6 cells, was associated with the induction of the insulin signaling pathway and glucose-stimulated insulin release. GP inhibition is also associated with β cell expansion in murine islets of Langerhans [12]. GP inhibitors induced the InsR/PI3K/AKT pathway and its downstream effectors, such as mTORC1 and mTORC2, indicated by enhanced phosphorylation of S6K and AKT. In the same study [12], we reported an increase in PDX1 and insulin levels that can be linked to the activation of the insulin signaling pathway. In the present study, BF142 treatment in MIN6 cells did not influence the activity of InsR and AKT, suggesting that the mechanism of BF142 action differs from previously tested compounds, and the effects are not connected with insulin signaling.

Similarly to our previous results [12], BF142 induced the activation of S6K protein, a key target protein of mTORC1. Phosphorylated S6K activates protein S6 of the ribosomal 40S subunit, inducing mRNA translation of ribosomal proteins, and, thus, increasing the cellular capacity for protein synthesis. Another target protein of mTORC1 is 4E-BP1 that also induces protein translation [46]. Therefore, mTORC1 activity likely contributes to the increased insulin protein levels observed in BF142-treated MIN6 cells. Elevated levels of insulin protein were confirmed by significantly increased insulin secretion. Mice with overexpressed S6K protein in their pancreatic islets showed better glucose tolerance due to increased insulin secretion [47]. PDX1 is crucial for all aspects of β cell structure and function, and for the expression of genes involved in glucose sensing and metabolism, mitochondrial function, and insulin secretion [48–50].

Growing evidence indicates that mTORC1 is critical for β cell expansion, growth, proliferation, and regeneration [51–55], as well as for β cell adaptation to hyperglycemia and insulin resistance [53]. Several studies confirm that the mTOR signaling pathway is deregulated in human diseases, including T2DM [56–58]. Blandino-Rosano *et al*. [59] revealed the indisputable role of endogenous mTORC1 signaling in β cells using knock-out mice (*βraKO*). *Raptor* deletion resulted in β cell failure and diabetes via reduced proliferation, cell size, cell survival, and insulin secretion [60]. Activation of mTORC1 can be mediated by intracellular signals triggered by growth factors, nutrients, and energy. In our case, the mTORC1 induction could be a consequence of elevated ATP or insulin levels observed after BF142 treatment. Other researchers suggest that the role of glucose and amino acids in the activation of mTORC1 is mediated by an increase in mitochondrial metabolism, even in MIN6 cells. Several studies confirm the link between mTOR signaling, mitochondrial activity, and insulin production [46, 61, 62]. Nevertheless, to determine the exact molecular pathways that bring about the effects of BF142 requires further investigations.

In our previous studies, GP inhibitors increased mitochondrial oxidation [12, 35]. BF142 did not enhance the oxygen consumption rate in MIN6 cells to the extent expected, but to a sufficient extent to significantly increase ATP levels and Ca^2+^-influx. Interestingly, the increase in glucose-stimulated insulin secretion lagged behind, and may be related to the lack of AKT activation or InsR signaling.

BF142, a tartaric acid derivative GP inhibitor, is far less potent than glucose derivatives (e.g. KB228) or indol-2-carboxamide (CP-316819) inhibitors [12]. Nevertheless, there are common points, including higher glycogen content, enhanced mitochondrial oxidation, Ca^2+^-influx, and induced mTORC1 activity or increased protein levels of PDX1 and insulin. As GP inhibitors with different chemical structure bring about similar biological effects, these data further confirm the involvement of GP and glycogen in the regulation of β cell function.

In our current study, the effects of an FR258900 analog GP inhibitor, BF142, was investigated in the MIN6 cell line. BF142 induced changes that supported β cell function similar to our previous observations with structurally different inhibitors [12, 35]. BF142 induced mitochondrial activity, elevated ATP production, Ca^2+^-influx, increased PDX1 levels, insulin production, and mTORC1 activation in a model of β cells. Our data confirm the importance of glycogen metabolism and mTOR signaling in supporting β cells.

## Supporting information

S1 Fig Uncut sample blots 1. **(A-C)** Proteins from Min6 protein lysates were separated by SDS-PAGE and were subjected to Western blotting. Membranes were probed with the antibodies indicated.
(PDF)

S2 Fig Uncut sample blots 2. **(A-F)** Proteins from Min6 protein lysates were separated by SDS-PAGE and were subjected to Western blotting. Membranes were probed with the antibodies indicated.
(PDF)
