# Supplementary figures and images for: Glycogen phosphorylase inhibitor, 2,3‐bis[(2E)‐3‐(4‐hydroxyphenyl)prop‐2‐enamido] butanedioic acid (BF142), improves baseline insulin secretion of MIN6 insulinoma cells
Source: PLoS One. 2020 Sep 22;15(9):e0236081. doi: 10.1371/journal.pone.0236081 (PMC7508380; doi:10.1371/journal.pone.0236081)

Supplementary figure 2.

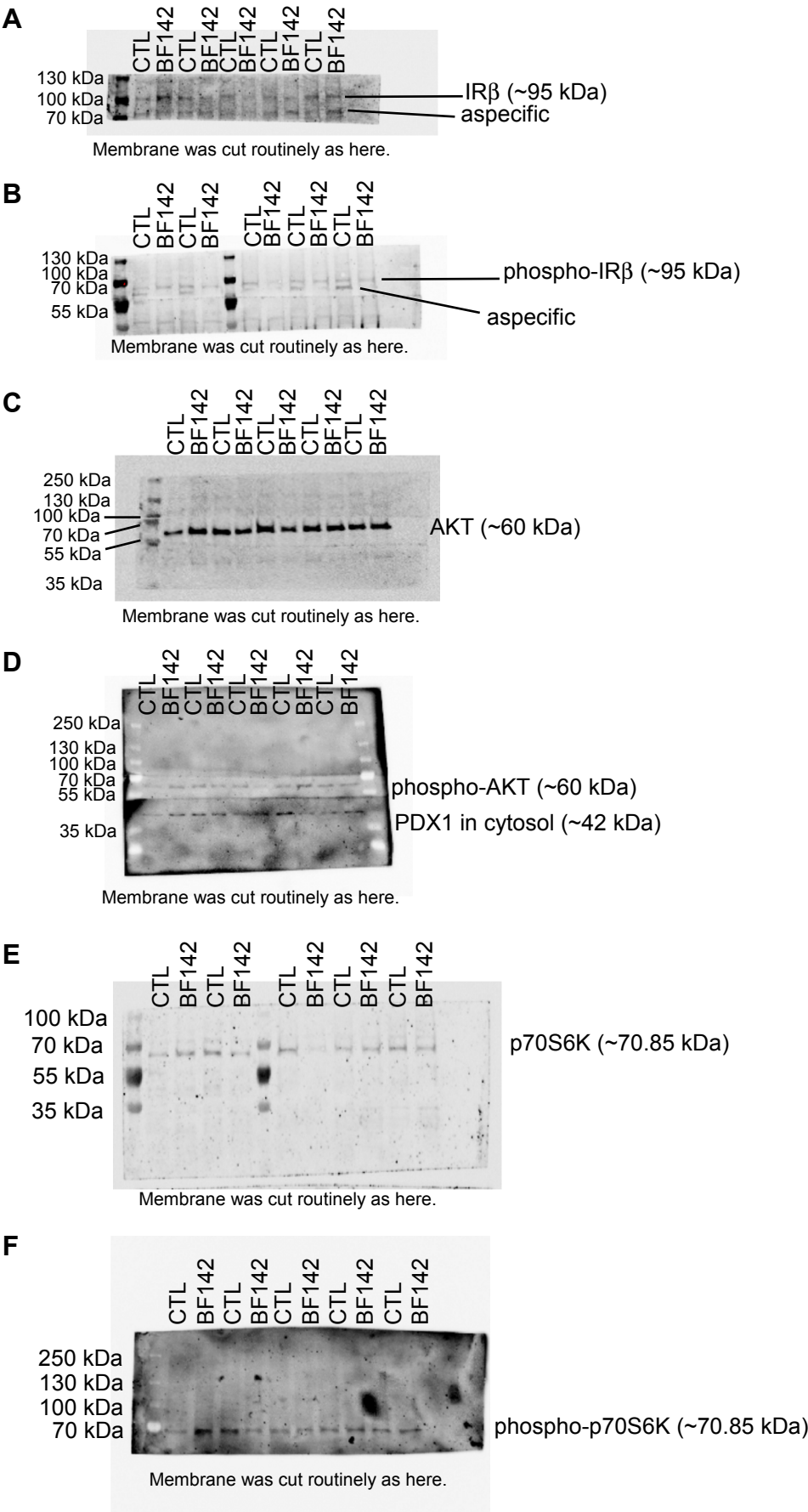

Supplement: S2 Fig — (A-F) Proteins from Min6 protein lysates were separated by SDS-PAGE and were subjected to Western blotting. Membranes were probed with the antibodies indicated. (PDF) [file pone.0236081.s002.pdf]
